# Peripheral B Lymphocyte Serves as a Reservoir for the Persistently Covert Infection of Mandarin Fish *Siniperca chuatsi* Ranavirus

**DOI:** 10.3390/v16121895

**Published:** 2024-12-09

**Authors:** Wenfeng Zhang, Hui Gong, Qianqian Sun, Yuting Fu, Xiaosi Wu, Hengwei Deng, Shaoping Weng, Jianguo He, Chuanfu Dong

**Affiliations:** 1 State Key Laboratory of Biocontrol, School of Life Sciences, Sun Yat-sen University, Guangzhou 510275, China; zhangwf27@mail.sysu.edu.cn (W.Z.); sunqq8@mail2.sysu.edu.cn (Q.S.); fuyt23@mail.sysu.edu.cn (Y.F.); wuxs6@mail2.sysu.edu.cn (X.W.); lsswsp@mail.sysu.edu.cn (S.W.); lsshjg@mail.sysu.edu.cn (J.H.); 2 Southern Marine Science and Engineering Guangdong Laboratory (Zhuhai), Zhuhai 519000, China; 3 Guangdong Province Key Laboratory of Aquatic Economic Animals, Institute of Aquatic Economic Animals, Sun Yat-sen University, Guangzhou 510275, China; 4 Fish Diseases Unit, Institute of Biotechnology, Fujian Academy of Agricultural Sciences, Fuzhou 350003, China; ghxfjm@163.com; 5 School of Marine Biology and Fisheries, Hainan University, Haikou 570228, China; hengweideng@163.com

**Keywords:** mandarin fish ranavirus (MRV), persistent infection, B lymphocytes, reactivation, TLR

## Abstract

Mandarin fish ranavirus (MRV) is a distinctive member among the genus *Ranavirus* of the family *Iridoviridae*. The persistently covert infection of MRV was previously observed in a natural outbreak of MRV, but the underlying mechanism remains unclear. Here, we show that mandarin fish peripheral B lymphocytes are implemented as viral reservoirs to maintain the persistent infection. When mandarin fish were infected with a sublethal dosage of MRV under a nonpermissive temperature (19 °C) and a permissive temperature (26 °C), all of the fish in the 19 °C group survived and entered the persistent phase of infection, characterized by a very low viral load in white blood cells, whereas some of the fish died of MRV infection in the 26 °C group, and the survival fish then initiated a persistent infection status. Raising the temperature, vaccination and dexamethasone treatment can reactivate the quiescent MRV to replicate and result in partial mortality. The viral reservoir investigation showed that IgM^+^-labeled B lymphocytes, but not CD3Δ^+^-labeled T lymphocytes and MRC-1^+^-labeled macrophages, are target cells for the persistent infection of MRV. Moreover, the reactivation of the quiescent MRV was confirmed through a non-TLR5 signal pathway manner. Collectively, we demonstrate the presence of the B cell-dependent persistent infection of ranavirus, and provide a new clue for better understanding the complex infection mechanism of vertebrate iridovirus.

## 1. Introduction

Iridovirus is a large viral family with a linear double-stranded DNA (dsDNA), genome sizes ranging from 99 kb to 220 kb and encoding open reading frames (ORFs) ranging from 98 to 221 [1,2,3,4]. The *Iridoviridae* family can be divided into two subfamilies, namely, *Alphairidovirinae* and *Betairidovirinae,* which are also referred to as vertebrate iridovirus (VI) and invertebrate iridovirus (IVI), respectively [2]. Among *Alphairidovirinae*, the *Ranavirus* genus has the most abundant members, with great diversity in the genome content, the most diverse array of hosts, including bony fish, reptiles and amphibians, and a very complex pathogenesis [5,6,7]. Ranavirus-associated viral diseases have caused considerable economic losses and ecological disasters worldwide due to their high contagiousness and lethality to various economically valuable bony fish species [6,7,8].

Mandarin fish ranavirus (MRV), also known as a variant of largemouth bass virus (LMBV) in the Santee–Cooper ranavirus (SCRaV) species of the genus *Ranavirus*, has emerged as an important pathogenic threat to several bony fish species. The known sensitive natural hosts of MRV/LMBV include largemouth bass (*Micropterus salmoides*) [9,10], koi carp (*Cyprinus carpio*) [11], barcoo grunter (*Scortum barcoo*) [12], two tropical ornamental fish, Doctor fish (*Labroides dimidatus*) and Guppy (*Poecilia reticulata*) [13], as well as mandarin fish (*Siniperca chuatsi*) [3,14,15]. In mandarin fish, MRV infection generally manifests in the following two distinctive clinical phenotypes: acute and chronic [4,14,15]. During acute infection, morbidity generally occurs at 2–4 days post-infection, and is characterized by vomiting and then severe ascites syndrome [15]. Subsequently, mass mortalities ensue within 3–10 days, with an accumulative mortality of >50% [14,15]. The tropism tissues during acute infection include the pyloric caecum, spleens, kidneys, livers and intestines [4,14]. In contrast, the chronic phase of infection is characterized by lethargy, anorexia, abnormal swimming and impaired coordination, and can persist for up to 5 months [14]. During chronic infection, most of the surviving fish become emaciated and deformed due to long-term anorexia or abnormal feeding, and then lose their breeding value. Especially during chronic infection, very low numbers of copies of viral genomic DNA were detected using real-time quantitative PCR (qRT-PCR), but not by conventional PCR, using the visceral organs such as the spleen, liver and kidney. We previously designated this long-lasting, undetectable status of the MRV carrier by using conventional PCR detection as the recessive carrier [14], and we currently designate it as a persistently covert infection (PCI). During MRV-PCI, sporadic death, but not mass mortality, are the major events.

Prior to the emerging MRV, infectious spleen and kidney necrosis virus (ISKNV), the type species in the genus *Megalocytivirus of Alphairidovirinae*, has been considered as the first important pathogenic threat to the mandarin fish industry [16]. Until recently, an inactivated ISKNV vaccine with an absolute protection effect of >90% was developed and officially licensed for a commercial application in mainland China [14,17]. The inactivated ISKNV vaccine also showed the same high efficacy in Asia seabass *Lates calcarifer* and spotted seabass *L. maculatus* against various genotypes of ISKNV/RSIV isolates in ISKNV species [18,19]. Unexpectedly, we attempted to develop an effective MFF-1 cell-based MRV vaccine, conducted with the same rational technology routine as that of the inactivated ISKNV vaccine, but failed. Almost no protection was achieved by the inactivated MRV vaccine, regardless of the MRV antigen dosage (unpublished data by Dong’s lab). Generally, ISKNV/RSIV is proposed to be a transient pathogen. As a result, either the inactivated vaccine or non-lethal active virus exposure could elicit robust immune protection against infection or re-infection with virulent ISKNV/RSIV [17,20,21,22]. Conversely, MRV displays the characteristics of persistent infection. As an opposite result, the inactivated MRV vaccine confers no protection. Additionally, the non-lethal active MRV exposure could cause long-term MRV-PCI [14].

White blood cells (WBCs), which have a pivotal role in the immune system, serve as dormant or latent carriers of numerous viruses. For instance, the human immunodeficiency virus (HIV) specifically targets T cells as its latent host cells, while the human T cell leukemia virus (HTLV) can establish residence in both B and T cells [23,24]. Similarly, the herpesvirus can conceal itself within B lymphocytes in both humans and animals [25,26,27,28,29]. These viruses strategically target the immune system for infection and latency, enabling them to elude immune clearance and persist within the host for extended periods. Studies have underscored the significance of macrophages (M_Ø_) as persistent hosts for amphibian ranaviruses [30,31,32,33]. FV3 is believed to undergo quiescent infection, which is evident in the detection of FV3 DNA within peritoneal leukocytes following the initial infection [34,35]. During the quiescent state, only inactive viral particles are identified within the peritoneal M_Ø_ [36]. The injection of heat-inactivated *E. coli* can reactivate FV3 in asymptomatic adult frogs and lead to fatal infections [37,38]. Besides FV3, no other ranaviruses have been studied comprehensively for persistent infection and reactivation. As a distinctive ranaviral member, here, we reveal that MRV has the unique characteristic of B cell-targeted persistent infection, which is not only completely different from that of the well-studied megalocytiviral ISKNV/RSIV but also is quite different from that of ranaviral FV3.

## 2. Materials and Methods

### 2.1. Animals, Virus and Antibodies

Three-month-old mandarin fish with a body weight of 200 g~300 g per fish were obtained from a fish farm in Foshan, Guangdong Province, China. All of the animal experiments were conducted in accordance with the animal testing procedures of Guangdong Province, China, and approved by the Ethics Committee of Sun Yat-sen University (approval number: SYSU-LS-IACUC-2024-0016). Before the experiment, the mandarin fish were randomly sampled and tested to confirm that they were free of asymptomatic MRV carriers by using qPCR. The MRV isolate ZQ17 was isolated and characterized from a mass mortality event of mandarin fish [14]. The mandarin fish fry (MFF-1) cell line was grown in complete Dulbecco’s modified Eagle’s medium (DMEM) (Gibco, Carlsbad, CA, USA) with 10% fetal bovine serum (FBS) (Gibco) at 26 °C, and also containing 5% CO_2_, so to amplify the MRV [39]. Anti-mouse monoclonal antibody (mAb) of MRV 1C4 was constructed in our recent report [4]. Anti-mouse mAb of mandarin fish IgM 7F12F6 was kept in our lab [40]. Polyclonal antibodies (pAbs) specific to the paired domain of the mandarin fish Pax5, specific to the mandarin fish T cell surface glycoprotein CD3 delta chain and specific to the mandarin fish mannose receptor C-type 1 were prepared and stored in our laboratory.

### 2.2. Microchip Label and Artificial Infection

Mandarin fish were divided into two groups. Group I was comprised of 15 fish reared in an aquatic environment with a constant water temperature of 26 °C. Group II consisted of 20 fish maintained in a water temperature environment of 19 °C. Prior to infection, the individual mandarin fish in group II were labeled as M1~M20 by the injection of a microchip into the back muscle. Following two weeks of acclimatization, all of the fish were challenged by intraperitoneal injection (i.p.) with MRV-ZQ17 at 10^6.5^ TCID_50_/0.1 mL/fish. Daily monitoring of morbidity and mortality was conducted over the subsequent months. The fish were fed with a daily diet of live commercial bait fish.

### 2.3. Raising Temperature Stress

To determine whether the quiescent MRV can be reactivated by raising the temperature stress, at 38 days post-infection (dpi), the water temperature of group II was incrementally increased from 19 °C to 26 °C, with a gradual rate of 1 °C per 2 days, ultimately maintaining a constant temperature of 26 °C until the end of the experiment. Subsequent to the temperature elevation, daily observation was conducted to monitor the pathological changes and mortality. The livers, spleens, kidneys and intestines from the diseased fish were collected for the qPCR analysis to determine the viral load. Additionally, MFF-1 cells were inoculated to assess the development of the cytopathic effects (CPEs). Concurrently, the fixed tissues were subjected to immunohistochemical analysis. When the temperature had been sustained at 26 °C for 5 days (at 57 dpi since the initial infection), the blood samples were obtained from the surviving fish through the tail vein following anesthesia for the purpose of WBC collection for the MRV load detection.

### 2.4. Vaccination, Dexamethasone (DXMS) Treatment and E. coil Stimulation

The MRV-ZQ17 isolate was used for the vaccine preparation, as previously described [41]. Briefly, confluent MFF-1 cells were inoculated with MRV with a multiplicity of infection (MOI) of 0.1. When the CPE was complete, at about 3 dpi, whole-cell suspensions were harvested and inactivated by formalin to a final concentration of 0.1% (*v*/*v*) at 4 °C for 10 days. The inactivated virus was emulsified with a water-in-oil (w/o) formulation with injectable white oil (Esso, Dole, France). *E. coli* cells (DH5&alpha) were cultured overnight at 37 °C, boiled for 1 h, pelleted by centrifugation, resuspended in PBS and adjusted to a final concentration to 500,000 cells/mL. Finally, the MRV-PCI mandarin fish were injected by i.p. with 100 μL of the MRV vaccine, 5.0 mg/kg of dexamethasone (DXMS) or 100 μL of the resuspended heat-killed *E. coil*, respectively. During these treatments, tissues from moribund fish were collected for viral load measurements.

To validate these findings, a replication experiment was conducted wherein fish with identical specifications were maintained at a temperature of 26 °C and infected with MRV by i.p. injection with 10^6.5^ TCID_50_/0.1 mL/fish. Subsequently, the survival fish were reared for 30 days. A qPCR was conducted to confirm that the survival fishes had entered into a persistent infection status, and then vaccination and DXMS treatments were exerted. During these treatments, tissues from the moribund fish were sampled for viral load measurements.

### 2.5. IgM^+^, CD3^+^ and MRC1^+^ WBC Isolation and Purification

WBCs were collected after layering whole blood on a Ficoll–Paque Plus gradient according to the manufacturer’s instructions (GE Healthcare, Pittsburgh, PA, USA) and washed twice with Hanks’ balanced salt solution (HBSS). Total WBCs were firstly stained with anti-mandarin fish IgM, CD3delta or MRC1 antibody on ice for 60 min, and rinsed twice with HBSS. The WBCs were then stained with secondary Alexa Fluor 488-labeled goat anti-mouse or rabbit IgG antibody with a 1:500 dilution at 4 °C for 60 min, and then washed twice, followed by positive and negative cell isolation using Beckman MoFlo Astrios EQs, according to manufacturer’s instructions (Beckman, Miami, FL, USA).

### 2.6. Western Blotting

The isolated WBCs were lysed using RIPA lysis buffer (Pierce, Waltham, MA, USA) and then subjected to 12% SDS-PAGE separation. The fractioned proteins were transferred for 90 min at 200 mA to nitrocellulose membranes (GE, Pittsburgh, PA, USA). The mAb of the mouse anti-mandarin fish IgM 7F12F6 and the pAbs of the rabbit anti-mandarin fish Pax5, CD3 and MRC1 were used as the first antibodies (Abs). The horseradish peroxidase (HRP)-labeled goat anti-mouse or goat anti-rabbit IgG (Sigma, shanghai, China) was used as the secondary Ab. The recognized protein bands were visualized by 3,3 N-Diaminobenzidine tetrahydrochloride (DAB) solution staining (Roche, Basel, Switzerland).

### 2.7. Confocal Microscopy Observation

The purified IgM^+^ cells and IgM^−^ cell residuals were fixed with 4% paraformaldehyde on tissue slides and permeabilized with 0.1% Triton X-100 in phosphate-buffered saline (PBS) containing 1% bovine serum albumin (BSA). The cells were stained with primary anti-IgM 7F12F6 mAb and anti-Pax5 pAb at 1:1000 dilutions at room temperature for 1 h, and rinsed twice with PBS. The cells were then stained with Alexa Fluor 565-labeled monkey anti-mouse IgG antibody and Alexa Fluor 488-labeled goat anti-rabbit IgG antibody at a 1:1000 dilution. Finally, the nucleus was stained by 4′, 6-diamidino-2-phenylindole (DAPI) (Abcam, Cambridge, UK). Sections were visualized under confocal laser scanning immunofluorescence microscopy (Leica SP8, Solms, German).

### 2.8. Viral Load Measuement by qPCR

The absolute qPCR was conducted to determine the MRV genome DNA copies using DNA templates isolated from WBCs or purified B, T, M_Ø_ and residual WBCs through FastPure Cell/Tissue DNA Isolation Mini Kit (Vazyme, Nanjing, China) according to the manufacturer’s instructions. A standard curve of the MRV MCP-specific qPCR system was established using a 10-fold serially diluted pMD-19T-MCP recombinant plasmid DNA (10^9^ copies/μL–10^1^ copies/μL). The MRV specific primer set used in this study targeted a partial fragment of the MCP gene, as shown in Table 1. The reactions were performed using the Light Cycler^®^ 480-II Multiwell Plate 384 real-time detection system (Roche Diagnosis, Basel, Switzerland) under the following conditions: 1 cycle at 95 °C for 60 s and 40 cycles of 95 °C for 5 s, and 60 °C for 30 s and 72 °C for 5 s. A non-template control was used as a negative amplification control. All of the qRT-PCRs were performed in triplicate.

### 2.9. Quantitative Gene Expression

The total RNA was extracted from the WBCs by using the Eastep Super Total RNA Extraction Kit (Promega, Shanghai, China) according to the manufacturer’ s instructions. Then, the RNA was reverse-transcribed into the cDNAs using Evo M-MLV RT Premix for qPCR (Accurate Biology, Shanghai, China). The expression levels of the MRV MCP gene and 16 different TLRs in the cDNA samples were quantified by qRT-PCR methods in the Roche LightCycler 480 system, with mandarin fish β-actin as the reference gene. Primers specific for 16 different TLRs were designed and validated by the gradient PCR and the qPCR melting curves. All of the primers used in the study are listed in Table 1. The qRT-PCR was performed as described previously [14]. Briefly, the PCRs were performed with the following procedure: 95 °C for 30 s—1 cycle; 95 °C for 5 s and 60 °C for 30 s—40 cycles; 95 °C for 5 s, 60 °C for 1 min and 95 °C—1 cycle; 50 °C for 30 s—1 cycle, with a total reaction volume of 10 μL, containing 1 μL of cDNA, 5 μL of 2 × SYBR Green Pro Taq HS Premix (Accurate Biology, Shanghai, China), 0.5 μM of primers (Tsingke, Beijing, China) and 3 μL of RNase-free water.

### 2.10. Immunohistochemistry Assay (IHC)

The tissues of moribund fish from the treatment with temperature stress, vaccination or dexamethasone stimulation were collected and fixed with alcohol–formal acetic (AFA) for IHC analysis as our previous description [14]. Briefly, intestines or pyloric caeca were dissected and fixed using AFA and then embedded in paraffin wax. The embedded tissues then were excised into sections of 4 μm in thickness. The sections were dewaxed in xylene and rehydrated in a series of ethanol. Positive sections were performed using anti-MRV mAb 1C4 (1:1000) as the primary antibody and horseradish peroxidase (HRP)-labeled goat anti-mouse IgG as the second antibody for the IHC analysis. Finally, the tissue sections were developed with DAB solution and visualized under a Nikon fluorescence microscope (Eclipse Ni-E, Tokyo, Japan).

### 2.11. Statistical Analysis

An analysis of variance (ANOVA) was performed for the statistical analysis of the expression and viral load data. The statistical analysis of the survival data was performed using a Log-Rank Test (GraphPad Prism 8, San Diego, CA, USA). A probability value of *p* < 0.05 was used in all of the analyses to indicate significance. Error bars on all of the graphs represent the standard error of the mean (SEM).

## 3. Results

### 3.1. Transition of MRV from Acute Infection to Persistently Covert State

**Figure 1:**
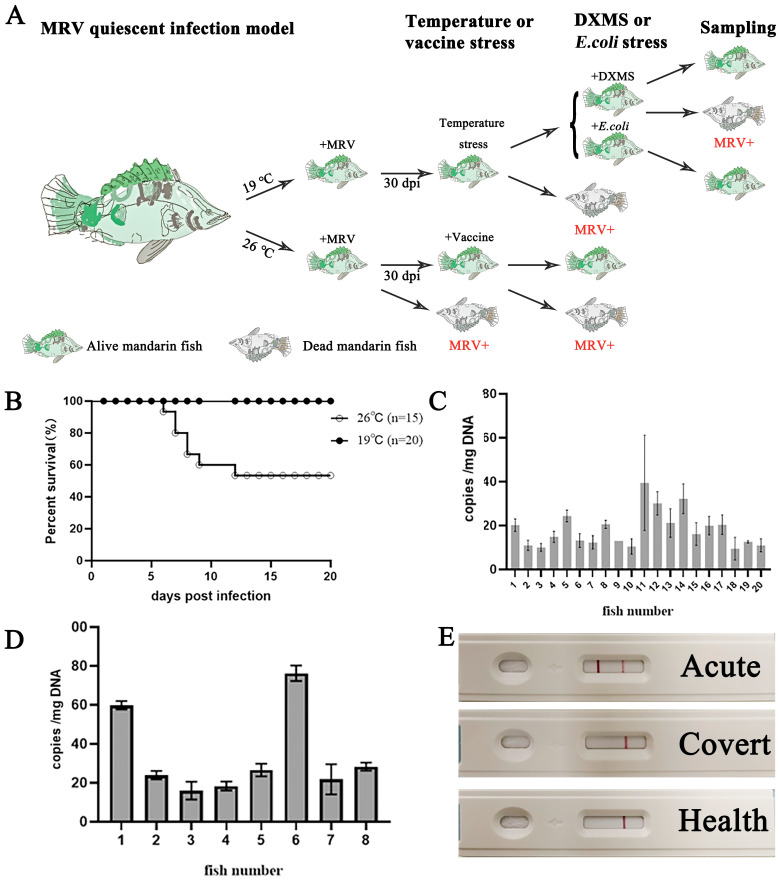
Tracking the status of MRV-infected mandarin fish under different temperature conditions. (**A**) The experimental outline of MRV infection and the quiescent model. Briefly, mandarin fish were infected under two different temperature conditions. In the 19 °C infection group, all of the fish survived to 30 dpi until raising the temperature stress. After acclimatization, the survival fish were treated with DXMS and heat-killed *E. coil*, respectively. In the 26 °C infection group, partial mortality occurred within 12 dpi. The survival fish were vaccinated with the inactivated MRV vaccine. (**B**) The survival curves of the MRV-infected mandarin fish under 19 °C and 26 °C. All of the fish survived in the 19 °C infection group, and partial mortality was observed in the 26 °C infection group. (**C**,**D**) The MRV copy number per mg WBC DNA isolated from the 20 mandarin fish in the 19 °C group and the 8 survival fish in the 26 °C group at 30 dpi, respectively. (**E**) Detection of MRV using the anti-MRV mAb-based colloidal gold-immunochromatographic fast-detection strip. A positive reaction was obtained only from the dead fish suffering from the primary infection of MRV in the 26 °C infection group (Acute), but negative for the 19 °C infection group and mock infection.

Two groups of mandarin fish reared in two different temperatures were challenged with a sublethal dose (10^6.5^ TCID_50_/fish) of MRV-ZQ17. As a result, the infected fish in the 26 °C group died at 6 dpi until there was no additional death occurrence at 12 dpi, with an accumulated mortality of 46.67% (7/15) (Figure 1B). The dead fish were confirmed as dying from MRV infection by conventional PCR detection. By contrast, no mortality was observed in the 19 °C group (Figure 1B). Observation was continued for thirty days, and no additional death occurred in both groups. At 30 dpi, blood was pooled from all survival fish to isolate the WBCs for the MRV genome DNA detection, and no MRV was detected by conventional PCR, whereas low copies of the MRV genome DNA were obtained from all 28 fish by absolute qPCR. The average viral loads in the WBCs from the 20 mandarin fish reared at 19 °C was 18.5 ± 9.7 genome equivalents (GEs) per mg DNA (Figure 1C), whereas it was 33.9 ± 20.7 GEs per mg DNA from the 8 survival mandarin fish reared at 26 °C (Figure 1D). Additionally, using an anti-MRV mAb-based colloidal gold-immunochromatographic fast-detection strip, blood samples from the survival fish showed negative results, whereas the result was strongly positive for the ascites sample from the moribund mandarin fish in group I (Figure 1E).

### 3.2. Reactivation of Covert MRV via Raising Temperature Stress

**Figure 2:**
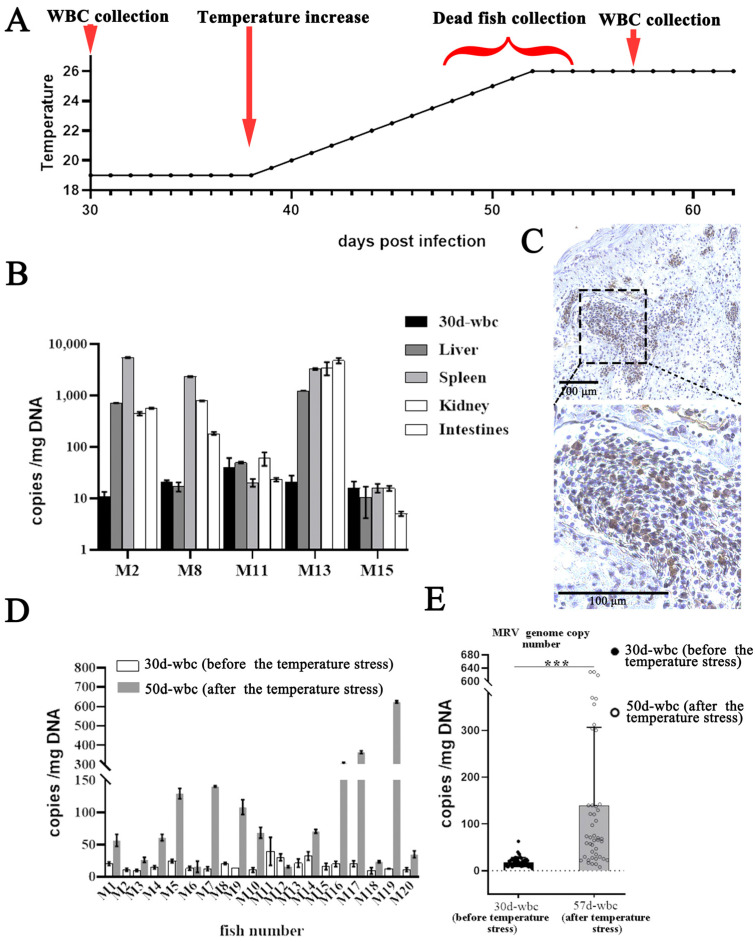
Reactivation of the covert MRV via raising the temperature stress. (**A**) The experimental outline of the reactivation of covert MRV via raising the temperature stress. Blood was drawn from the infected fish in the 19 °C group for the MRV load analysis at 30 dpi. After 7 days of acclimatation, at 38 dpi, the water temperature was gradually elevated, with 1 °C per 2 days. During the raising of the temperature stress, dead fish were sampled for MRV load measurements, and the survival fish were used for the second round of blood collection for the MRV load assessment. (**B**) The MRV DNA load was measured in various tissues from 5 dead fish during the raising of the temperature stress. High MRV loads were observed in M2, M8 and M13. (**C**) The IHC of the intestines of the dead fish M13. The tissue was recognized with mAb 1C4, and numerous infected cells were stained brown by DAB. The dashed box indicates the enlarged area. Bar = 100 μm. (**D**,**E**) The MRV copy number per mg WBC DNA of each (**D**) and the gross distribution (**E**), respectively. Fifteen survival mandarin fish on the 5th day after the completion of the raising temperature stress and the comparison with that before stimulation. ***, *p* < 0.001.

Following the initial blood drawing for the WBC separation at 30 dpi, the mandarin fish were allowed to recover for 7 days. Subsequently, the temperature stress was raised in 19 °C infection group. As a result, a total of five mandarin fish (designated as M2, M8, M11, M13 and M15) died successively on the 10th, 12th, 14th and 15th days (from 24 °C to 26 °C). Among them, obvious ascites was observed only in M13. Moreover, high levels of MRV load were measured in various organs of M2, M8 and M13 (Figure 2B), indicating that these fish died of MRV infection. IHC also demonstrated a substantial number of virus-positive signals, also indicating an active MRV infection (Figure 2C). After raising the temperature stress, the second round of blood collection was conducted at 57 dpi, and WBCs from the 15 surviving mandarin fish were collected (Figure 2A). The qPCR analysis revealed that the average MRV copy number in the WBCs from the 15 surviving mandarin fish was 139 ± 165 GEs/mg DNA (Figure 2D,E).

### 3.3. Reactivation of Covert MRV by Vaccination and DXMS Treatment

**Figure 3:**
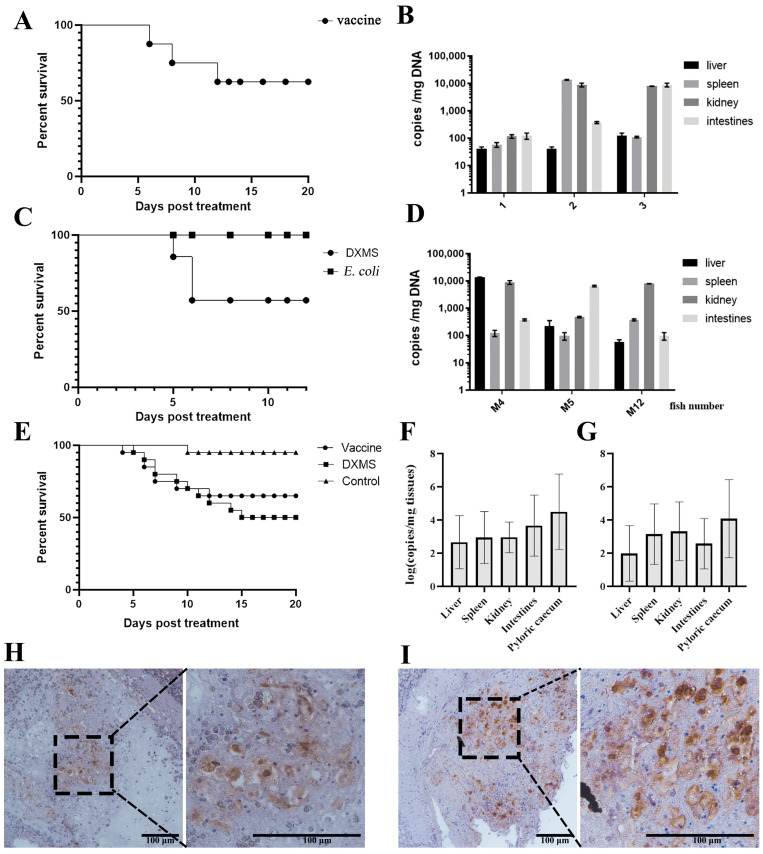
Assessment of the reactivation effects of covert MRV via vaccination, DXMS stimulation and *E. coli* injection. (**A**) Survival curve of the MRV-PCI mandarin fish via vaccination stimulation. (**B**) Tissue MRV DNA load examination of 3 dead fish suffering vaccination stimulation. (**C**) Survival curves of the MRV-PCI mandarin fish via DXMS treatment and *E. coli* injection. Three fish died during the DXMS treatment and no fish died during the *E. coil* injection. (**D**) Tissue MRV DNA load examination of 3 dead fish suffering from the DXMS treatment. (**E**–**G**) The repeated experiments (N = 20) of the assessment of the reactivation of MRV-PCI mandarin fish via vaccination and DXMS treatments, where 1, 7 and 10 fish died from the control, vaccination and DXMS treatments, respectively. High tissue MRV DNA loads were determined in dead fish from the vaccination (**F**) and DXMS treatment (**G**), respectively. Strong positive signals by IHC were also observed in the pyloric caeca from the vaccination (**H**) and DXMS treatment (**I**), respectively. The dashed box indicates the enlarged area. Bar = 100 μm.

To examine the effect of the inactivated vaccine on the reactivation of covert MRV, eight of the surviving mandarin fish from the 26 °C challenge (group I) were selected for the i.p. of 100 µL of inactivated MRV vaccine at 38 dpi. As a result, three fish succumbed on the 6th, 7th and 12th days post-vaccination (Figure 3A). QPCR analysis confirmed significant viral load changes in all three dead fish (Figure 3B), suggesting that the inactivated MRV vaccine reactivated the covert MRV and partially resulted in the fatal outcome. A repeated experiment involving 20 covert-infected individuals injected with the MRV vaccine resulted in an accumulative mortality of 35% (7/20) (Figure 3E). The QPCR of the tissues from the diseased fish showed high levels of MRV load (Figure 3F). Immunohistochemistry also exhibited a substantial number of virus-positive signals in the pyloric caeca (Figure 3H).

At 64 dpi, 14 of surviving mandarin fish in group II were divided into two groups, where 7 fish received an i.p. injection of 100 µL of DXMS, and another 7 fish were injected via i.p. with heat-inactivated *E. coli*. Daily observation revealed that three fish died on the 5th and 6th days post-DXMS treatment (M4, M5 and M12), respectively (Figure 3C). QPCR analysis confirmed the high viral loads in these dead fish (Figure 3D). A repeated experiment with 20 covert MRV mandarin fish treated with DXMS resulted in an accumulative mortality of 50% (10/20) (Figure 3E). The qPCR quantification of the tissues from the deceased fish showed high levels of MRV genome DNA (Figure 3G), and IHC also displayed a significant number of virus-positive signals in the pyloric caeca (Figure 3I). All of these results suggest DXMS’s ability to reactivate covert MRV to fatal outcomes. No dead fish were observed in the *E. coli* injection group (Figure 3C). Blood samples were collected from all of the survival fish on the 7th day post-*E. coli* treatment for conventional PCR or colloidal gold-immunochromatographic fast detection, and both showed MRV negativity, indicating that the *E. coli* treatment could not activate covert MRV to a detectable level.

### 3.4. Purification of B Cells, T Cells and M_Ø_

**Figure 4:**
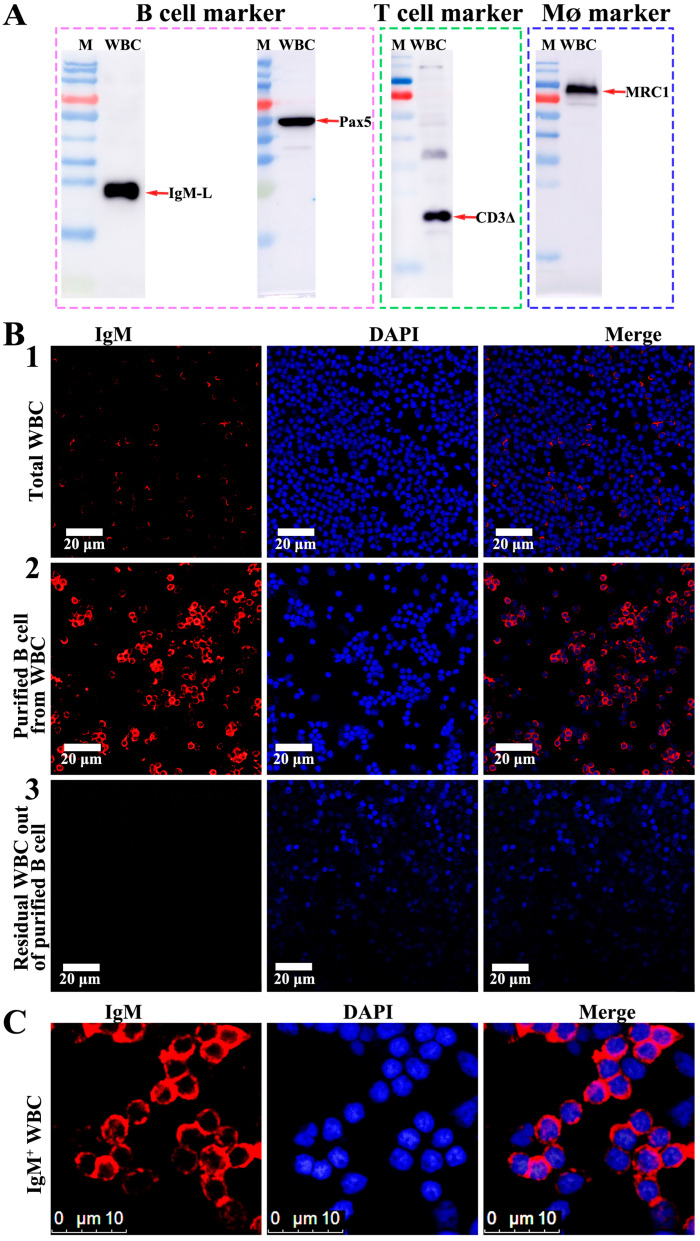
Detection of the antibody-targeted cell markers from mandarin fish WBCs. (**A**) Western blotting analysis of four cell markers of mandarin fish WBCs. The mAb of 7F12F6 recognized the light chain of the mandarin fish IgM. Three pAbs also recognized the corresponding protein bands, as speculated. (**B**,**C**) Confocal micrographs of mandarin fish WBCs. (**B1**) A few IgM^+^-labeled B cells from native WBCs. (**B2**) Numerous IgM^+^-labeled B cells after the flow cytometry sorting. (**B3**) Almost no IgM^+^-labeled B cells in the residual WBCS out of the B cells. Bar = 20 μm. (**C**) The enlarged image of the sorted IgM^+^-labeled B cells by the FACS. Bar =10 μm.

**Figure 5:**
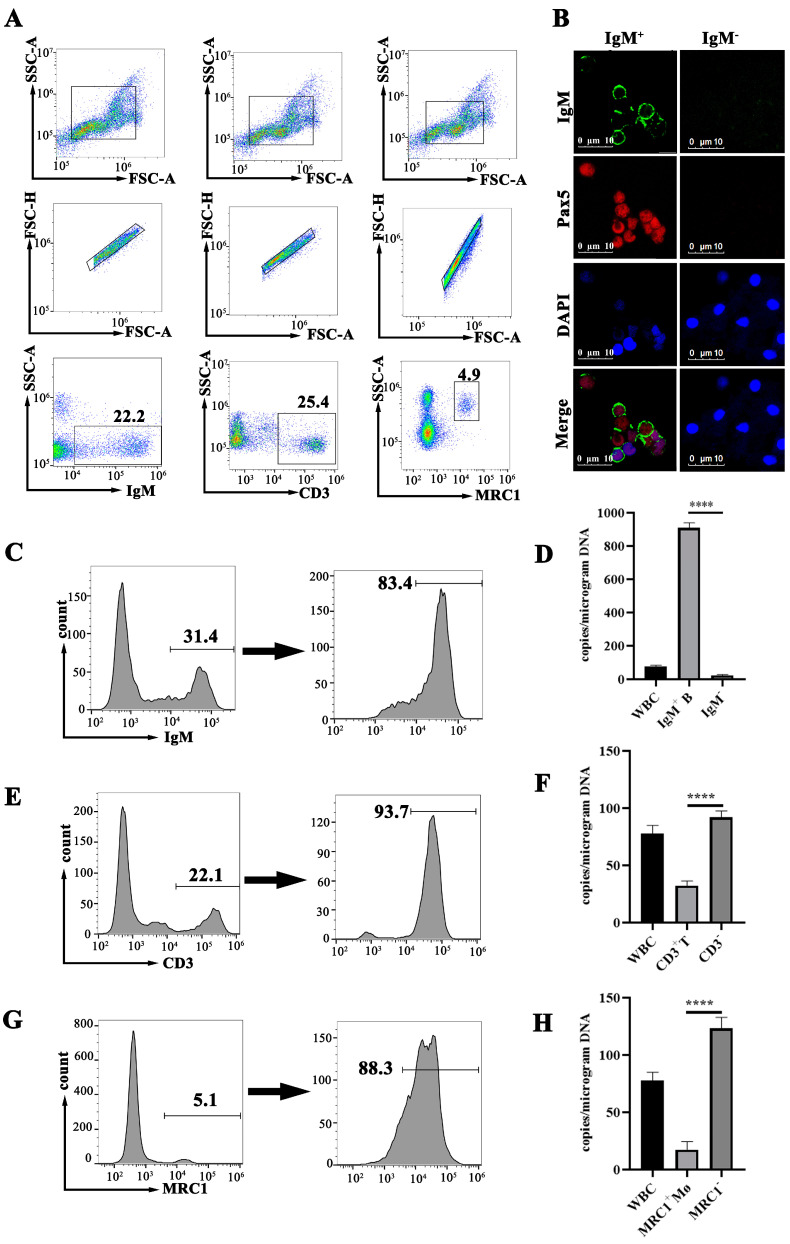
Fluorescence-activated cell staining (FACS) sorting of mandarin fish peripheral WBCs. (**A**) Flow cytometry of the mandarin fish WBCs stained with antibodies to the mandarin fish IgM (left), CD3 (middle) and MRC1 (right). (**B**) Confocal micrographs of the mandarin fish IgM^+^ and IgM^−^ WBCs. IgM^+^ cells were identified by anti-mandarin fish IgM mAb (7F12F6) and secondary Alexa Fluor555-labeled goat anti-mouse IgG (red). Pax5^+^ cells were identified by anti-Pax5 pAb and secondary Alexa Fluor488-labeled goat anti-rabbit IgG (green). The nucleus was identified with DAPI (blue). Bar = 10 μm. (**C**,**D**) show the progress of the IgM^+^ WBCs selected by the FACS and the copy number of the MRV genomes determined from 1 μg of DNA from the total WBCs, the selected IgM^+^ B cells and the residual IgM^−^ WBCs from the MRV-PCI mandarin fish, respectively. (**D**–**F**) The progress of the CD3^+^ WBCs selected by the FACS, and the copy number of the MRV genomes detected from 1 μg of WBC DNA, selected CD3^+^ T cells and CD3^−^ WBCs from the MRV-PCI mandarin fish, respectively. (**G**,**H**) The MRC1^+^ WBCs selected by the FACS (**G**) and the copy number of the MRV genomes from the WBCs, selected MRC1^+^ Mø and MRC1^−^ WBCs from the MRV-PCI mandarin fish, respectively. Statistical significance between the control and treated groups is denoted by ****, where the *p*-value was 0.0001, using a one-way ANOVA and Tukey’s post hoc test.

Flow cytometry was employed to screen and concentrate the B cells, T cells and M_Ø_ in the peripheral blood. The sorting markers included IgM and the transcription factor Pax5 (an additional marker specific to B cells) for the B cells, CD3Δ for the T cells and MRC1 for the M_Ø_. The validity of these cell markers was firstly conducted through Western blotting. The result showed that all four antibodies could recognize these markers from the WBCs in their expected positions (Figure 4A). The subsequent flow cytometry analyses demonstrated that, in the peripheral blood WBCs of the covert MRV-carried fish, the IgM^+^ B lymphocytes constituted approximately 25 ± 7.3%, the CD3Δ^+^ T lymphocytes accounted for around 24 ± 4.6% and the MRC1^+^ M_Ø_ made up about 5 ± 2.4% (Figure 5A). Following sorting by flow cytometry, the percentage of IgM^+^ B lymphocytes were increased from 31.4% to 83.4%, CD3Δ^+^ T lymphocytes were increased from 22.1% to 93.7% and MRC1^+^ M_Ø_ were increased from 5.1% to 88.3% (Figure 3E–G). The concentration process of B cells was assessed by an anti-IgM mAb-based and anti-Pax pAb-based immunofluorescence assay (IFA) by using confocal microscopy. As shown in Figure 4B, only a few WBCs could be stained by anti-IgM mAb in the native WBCs (Figure 4(B1)); however, the majority of the cells were recognized by the anti-IgM mAb after the FACS sorting (Figure 4(B2),C). By contrast, almost no fluorescence signal was observed in the residual WBCs from the isolation of the B cells (Figure 4(B3)). All of these results indicated that the B cells were well isolated and purified from the WBCs. Additionally, the co-expression of mIgM and Pax in the purified B cells was also observed (Figure 5B). In agreement with the FACS analysis, most of the flow cytometry-sorted IgM^+^ WBCs exhibited IgM staining on the cell surface, whereas the Pax5 staining localized in the nucleus (Figure 4B,C and Figure 5B).

### 3.5. MRV Genome Assessment in B Cells, T Cells and M_Ø_

To determine which WBC type had the target cell response for the covert MRV, the MRV genome copies in the purified B cells, T cells and M_Ø_ from the MRV-PCI mandarin fish were determined by qRT-PCR. As a result, under an equal amount of total template DNA, the MRV genome DNA load in the purified IgM^+^ B cells was about 40-fold more abundant than that in the IgM^−^ WBCs (Figure 5D). By contrast, the MRV genome loads in the CD3Δ^+^ T cells and in the MRC1^+^ M_Ø_ were only approximately one-third and one-tenth of those in the CD3Δ^−^ WBCs and MRC1^−^ WBCs, respectively (Figure 5F,H), clearly indicating that B lymphocytes, but not T lymphocytes and M_Ø_, were the targeted cells for the establishment of persistent MRV.

### 3.6. Screening TLR Pathway Involved in the Reactivation of Covert MRV

**Figure 6:**
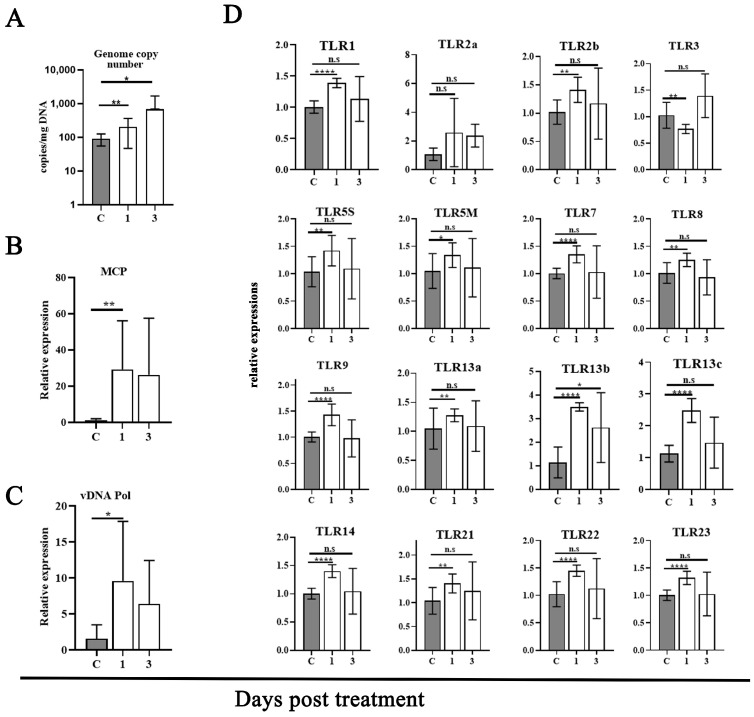
Differential expressions of TLR genes in the WBCs of MRV-PCI mandarin fish following DXMS stimulation. (**A**) MRV genome DNA copy numbers of the WBCs in the control, 1 day post-treatment and 3 days post-treatment. (**B**,**C**) Relative MCP gene and DNA polymerase gene expression in the control, 1 day post-treatment and 3 days post-treatment, respectively. (**D**) Relative gene expression levels of the 16 TLRs of the WBCs in the control, 1 day post-treatment and 3 days post-treatment. Statistical significance between the control and treated groups is denoted by *, where the *p*-value was 0.05, denoted by **, where the *p*-value was 0.01, and denoted by ****, where the *p*-value was 0.0001, using a one-way ANOVA and Tukey’s post hoc test. n.s., not significant. The gray bars indicate the control group, and the white bars indicate the treatment groups.

To investigate the possible involvement of a particular TLR pathway upon the reactivation of covert MRV, the differential expression of TLR genes during the transition of covert MRV to reactivation was assessed. Mandarin fish (weighing ~250 g) were infected with a sublethal dose (10^6.5^ TCID_50_/fish) of MRV and maintained for 30 days to ensure the establishment of MRV-PCI. At 30 dpi, the MRV-PCI mandarin fish were injected with DXMS per the above-mentioned approach. The injection of an equal amount of PBS was set as a control. The blood was pooled and the peripheral WBCs were isolated at 1 day and 3 days post-treatment. An absolute qPCR was conducted to determine the viral genomic load. Moreover, qRT-PCR was conducted to determine the transcription expression of viral MCP and DNA polymerase, as well as 16 different TLRs. As a result, the MRV genomic load in the WBCs increased significantly (*n* < 0.1) regardless of the DXMS treatment at 1 day and 3 days (Figure 6A). At the transcription level, the significant upregulation expression of viral MCP and DNA polymerase were also measured (Figure 6B,C), indicating the covert MRV was reactivated to replicate upon DXMS treatment. Meanwhile, all of the TLRs except for TLR2A showed inductive effects, with the expression increasing by less than 1.5 times (Figure 6D).

**Figure 7:**
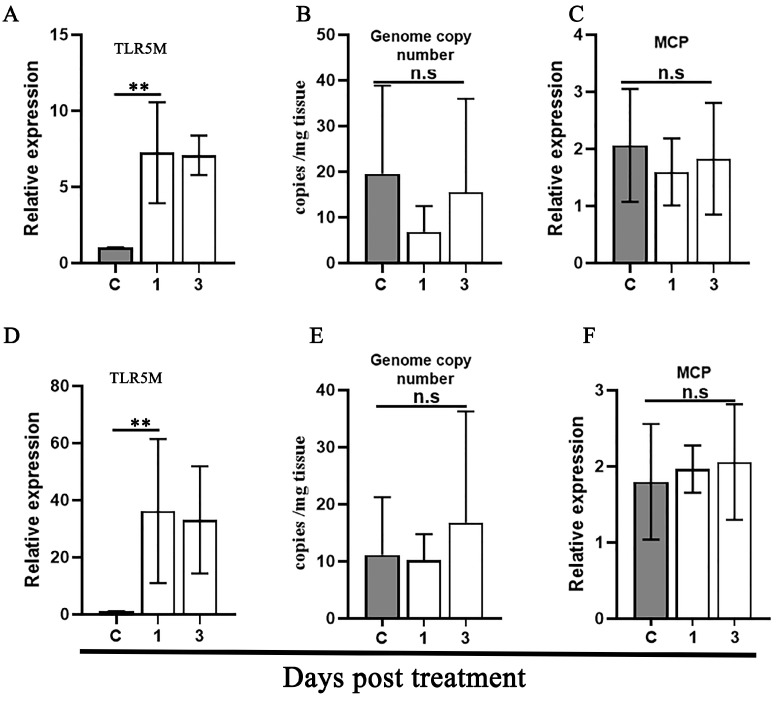
Assessment of the reactivation of the covert MRV and TLR5 signal by stimulation with *E. coli* and flagellin. Relative TLR5M gene expression (**A**), MRV genome copy number (**B**) and relative MCP expression (**C**) upon *E. coli* stimulation. Relative TLR5M gene expression (**D**), MRV genome copy number (**E**) and relative MCP expression (**F**) upon flagellin stimulation. Relative gene expression levels of the WBCs from MRV covert fish stimulated with *E. coli* and flagellin for 1 or 3 days, or injected with PBS as a control. Statistical significance between the control and treated groups is denoted by**, where the *p*-value was 0.01 using a one-way ANOVA and Tukey’s post hoc test. n.s, not significant. The gray columns indicate the control group, and the white columns indicate the treatment groups.

Given that *E. coli* and flagellate-stimulated TLR5 are implicated in the activation of quiescent FV3 infection [38], the homologous ligands were further used to determine the stimulation specificity of TLR5 in mandarin fish WBCs. As a result, the injection by i.p. of inactivated *E. coli* and flagellin both led to a sharp upregulation expression of TLR5M transcriptions in the WBCs at 1 and 3 dpi (Figure 7A,D). However, no increase in the MRV genome DNA copy and no upregulation expression of the MCP RNA were determined (Figure 7B–F), indicating that *E. coli* and flagellate stimulation initiated the TLR5 pathway, as expected; however, the active TLR5 was not linked to the reactivation of covert MRV.

## 4. Discussion

The natural persistent infection of MRV has been investigated and characterized in our previous report [14]. The core points of persistent MRV infection include the following: (a) it is a long-term chronic infection with a disease duration of up to 5 months, almost covering the entire breeding cycle of commercial mandarin fish; (b) during persistent infection, the MRV load in various visceral organs is too low to be detected by conventional PCR; (c) the featured clinical signs include lethargy, anorexia and body emaciation and deformity, accompanied with sporadic mortality. Furthermore, our recent study showed that the w/o formulation of the inactivated MRV vaccine conferred no protection against the parental virus challenge, which was also confirmed by several other research teams. Thus, the underlying mechanism of persistently covert infection and the ineffectiveness of inactivated vaccines have become the major issues currently troubling the understanding of MRV.

For cold-blooded animals, many habitat characteristics such as the water chemistry, soil type, ambient temperature, hydroperiod, UV-B as well as population density can affect the onset of viral diseases [7]. In the artificial challenge of ISKNV, a water temperature of 20 °C was experimentally evidenced as the threshold temperature [43]. Over 20 °C, the ISKNV was shown to be highly lethal to mandarin fish regardless of the various infection routines, including the administration by i.p. or intramuscular (i.m.) injection and cohabitation infection manner; however, ISKNV never showed lethality to mandarin fish when the experimental temperature was below 20 °C. In this study, our data showed that 19 °C was also a nonpermissive temperature for the onset of MRV disease, whereas 26 °C was a permissive temperature for MRV outbreak. Under a sublethal dosage of MRV challenge, no fish died (0/20) in the 19 °C group, but 46.7% (7/15) of the fish died of MRV infection, accompanied with ascites symptoms, within 5 dpi in the 26 °C group. The MRV could be easily detected through the colloidal gold-immunochromatographic fast-detection strip, conventional PCR as well as MFF-1-based virus isolation. At 30 dpi, the average viral loads in the WBCs of the 20 mandarin fish reared at 19 °C was 18.5 ± 9.7 GEs per mg DNA, whereas it was 33.9 ± 20.7 GEs per mg DNA from the 8 survival mandarin fish reared at 26 °C. Since no additional mortality occurred and the viral load in the blood was too low to be detected by conventional PCR and the colloidal gold-immunochromatographic fast-detection strip, it is proposed that all of these fish entered the covert infection phase. Furthermore, the result also suggested that MRV infection under the nonpermissive temperature of 19 °C could spontaneously result in the entry into the persistently covert infection status. By contrast, under the permissive temperature of 26 °C, the infection with a sublethal dosage of MRV could result in partial mortality, and the survival fish could then be transited into the persistent state.

During the gradual raising of the temperature, five fish died and three of them were measured with high viral loads. The absolute qPCR showed that the average viral load (N = 15) in the WBCs from the survival fish was 139 ± 165 GEs/mg DNA, which is much higher than that (18.5 ± 9.7 GEs per mg DNA) from the fish (N = 20) before the temperature stress. The result again indicated that raising the temperature stress could reactivate covert MRV to replicate and result in a fatal outcome, and then the survival fish once again entered into the persistent state of infection. After two rounds of drawing blood, and following another 7 days of stabilization, the surviving fish were divided into two groups and treated with DXMS and heat-killed *E. coil*, respectively. As a result, mortality was observed in the DXMS-treated group but not in the heat-killed *E. coil*-treated group. Both the viral load determinations and the IHC analysis demonstrated that DXMS functions as an effective inducer to trigger the reactivation of persistent MRV, which were confirmed by two independent repeated experiments in this study. DXMS, a classic glucocorticoid immunosuppressant, was commonly used in the reactivation of various viruses with persistent or latent characteristics [44,45,46]. For instance, cyprinid herpesvirus 2 (CyHV-2), a highly contagious pathogen of goldfish (*Carassius auratus*) and Prussian carp (*Carassius auratus gibelio*), establishes persistent infection in goldfish monocytes/M_Ø_. DXMS could induce the persistent CyHV-2 to replicate via effectively suppressing monocyte/M_Ø_ function and antibody production [44]. The mechanism of the DXMS-induced reactivation of covert MRV remained unclear and will be investigated in the future.

A previous study showed that vaccination with the w/o formulation of the inactivated ISKNV vaccine could induce the mass mortality of mandarin fish with covert MRV [14]. This study showed that the w/o formulation of the inactivated MRV vaccine also induced a similar outcome for covert MRV. It was proposed that, on the one hand, the white oil component played an adjuvant role in the w/o formulation of the emulsified vaccine, and, on the other hand, the obvious side effect of the w/o formulation of the vaccine is the induced local fibroperitonitis [47,48]. The induced peritonitis might trigger the reactivation of covert MRV. Generally, this study once again demonstrates the presence of a persistently covert infection of MRV in mandarin fish under laboratory conditions. Furthermore, the covert MRV can be triggered reactivation through various pathways, encompassing temperature stress, vaccination and the stimulation of the immunosuppressive agent DXMS.

Numerous epidemiological investigations and clinical data suggest that ranavirus has the capability to establish asymptomatic infections and reactivation under specific conditions [7,32,36,37]; however, the exact persistent infection has only been validated in FV3. In the case of FV3, the peritoneal M_Ø_ was confirmed as the major reservoir for quiescent FV3, and the quiescent FV3 can be reactivated by *E. coli* flagella-mediated TLR5 signal pathway [36,38]. In mammalian systems, human herpes simplex virus (HSV) establishes latency in ganglia, reactivating under reduced body resistance or various stress-induced host immune states [25]. Similarly, bovine herpesvirus 1 (BoHV-1) achieves latency in the spleen, tonsils and groin lymph nodes, reactivating after various stress stimulations [45], while γ-herpesviruses, including human Epstein–Barr virus (EBV) and murine γ-herpesvirus 68 (MHV-68), establish latent infection in B lymphocytes [49]. In fish, CyHV3/KHV establishes latency in the B lymphocytes of koi carp, reactivating in response to temperature stimulation and physical stress [26,50], whereas CyHV2/GFHNV establishes persistent infection in goldfish monocytes/M_Ø_ and could be reactivated by DXMS treatment [44]. In this study, we employed specific antibodies against MRC1 to isolate M_Ø_ and purified the T cells and B cells by using specific antibodies against CD3 and IgM. MRC1, a pattern-recognition receptor, serves as a marker for M_Ø_ and has been identified in various fish species [51,52]. CD3, exclusive to T cells, is composed of six peptide chains, and participates in T cell antigen recognition and signal transduction [53]. IgM, the principal component of fish immunoglobulins produced by B cells, functions as an antigen receptor on B lymphocytes. To determine the identity of the selected IgM^+^ cells, the antibody specific to the B cell transcription factor Pax5 was also used in our study. Pax5 is a major regulator of B cell development and has been identified in both mammalian and non-mammalian species [54,55]. Pax5 is mainly expressed in vertebrate B cell lineages [56], including bony fish species such as rainbow trout, puffer fish and zebrafish [57,58,59]. Furthermore, using these anti-mandarin fish marker antibodies, B cells, T cells and M_Ø_ were well isolated and purified from the WBCs. The qPCR showed that the MRV genomic DNA load in the IgM^+^ B cells was 40-fold higher than that in the IgM^−^ WBCs after isolation. By contrast, the MRV genomic loads in the purified CD3^+^ T cells and MRC1^+^ M_Ø_ were much lower than those in the MRV-PCI WBCs. All of these results clearly showed that peripheral IgM^+^ B lymphocytes, but not CD3^+^ T cells and MRC1^+^ M_Ø_, act as the primary target cells for the covert infection of MRV.

To assess the possible TLR signals involving the reactivation of covert MRV, 16 mandarin fish TLR molecules were screened. Under the DXMS treatment, the covert MRV was reactivated, as expected, with the slight upregulation expression of 15 of the 16 TLR genes. Meanwhile, when the MRV-PCI mandarin fish were injected with heat-killed *E. coli* or flagella, the TLR5 signal was significantly activated, as expected; however, the covert MRV was not reactivated. All of these results suggest that the reactivation of covert MRV occurs in a non-TLR5 manner, which is different from that of persistent FV3 [38]. The dissimilarity in their activation pathways may be attributed to their distinct taxonomic status and host preferences, despite both belonging to the *Ranavirus* genus within the *Iridoviridae* family. Specifically, ranaviruses can be classified into seven species, among which, FV3 in the ALRV subclass, primarily infecting amphibians and reptiles, and MRV in the SCRaV species and members in the GIV (grouper iridovirus) species only infect bony fish [5]. At the genomic level, the ALRV subclass genome is approximately 105 kb [1,60], the GIV genome is about 140 kb [61] while, by contrast, the MRV genome is only about 99 kb [3,4]. Notably, the homology of the MCP between MRV and other ALRV members is less than 84%. The genomic differences may contribute to the heterogeneity observed in fish ranavirus infections compared to that of amphibian ranavirus [3].

It is worth noting that the pre-exposure to virulent megalocytiviral ISKNV/RSIV in nonpermissive temperatures could elicit robust protective immunity against the re-challenges with parental viruses in permissive temperatures; thus, so-called live ISKNV/RSIV vaccines were developed on the basis of temperature regulation [22,62]. As for MRV, the low number of copies of the MRV genome DNA in the nonpermissive temperature of 19 °C maintained for at least 30 days. Even if a mild warming stimulation was applied, a partial mortality outcome occurred, and the survival retransformed into a persistent status. Finally, the covert MRV could once again be reactivated by the DXMS treatment. Obviously, the exposure to virulent live MRV under a nonpermissive temperature could not induce enough immune clearance, which is highly consistent with the field observation. In production practice, the surviving fishes from the ISKNV epidemic would usually never experience a recurrence of ISKNV infection; however, for MRV, after a primary infection, the surviving fishes were transformed into a chronic infection state, which could last for at least 5 months [14]. Whether the B cell targeting of persistent infection is a key factor affecting the activation of the immune clearance is worthy of further investigation. Specifically, in contrast to the highly effective inactivated ISKNV vaccine, robust experimental evidence shows that the w/o formulation of the inactivated MRV vaccine confers almost no protection against the challenge with virulent MRV regardless of the antigen dosage. Whether the ineffectiveness of inactivated vaccines is associated with the persistent targeting of MRV-specific B cells and its potential mechanisms will be an important topic in the study of its pathogenesis and the vaccine prevention of MRV.

## 5. Conclusions

In vertebrate iridovirus, FV3, the type species of genus *Ranavirus*, was evidenced to establish persistent infection by using *Xenopus* peritoneal M_Ø_ as reservoirs. MRV is a very distinctive ranavirus from FV3 with very different genomic content and host species. Here, we uncovered that MRV establishes persistent and covert infection by using peripheral B lymphocytes as virus reservoirs. During persistent infection, very low numbers of copies of quiescent MRV were harbored in peripheral B lymphocytes. Water temperature stress, vaccination stimulation and DXMS treatment can reactivate the quiescent MRV to replicate in abundance via a non-TLR5-mediated manner, and results in the recurrence of MRV disease. Our finding suggests the diversity and complexity of the pathogenic mechanisms among ranaviruses, and also has important scientific significance for the in-depth understanding of the infection and immunity interaction of vertebrate iridoviruses, especially ranavirus.

## Figures and Tables

**Table 1:** PCR prime sequence ^a^.

Genes	Primers	Sequences
MCP probe	Forward	5′-CACGCCGCACTCTCGTT-3′
	Reverse	5′-GCGTCCAGGAAAGCAGTGTT-3′
	Probe	5′-AACGAGATTCAGGCCCAG-3′
MCP	Forward	5′-TCGCCACTTATGACAGCCTTGA-3′
	Reverse	5′-CGGCACTGATGGCACTTGAC-3′
vDNA polymerase II	Forward	5′-TCTGCGTTAGGGTGACTGGTTT-3′
	Reverse	5′-CGGCACTGATGGCACTTGAC-3′
β-actin	Forward	5′-AGAGGGAAATCGTGCGTG-3′
	Reverse	5′-GAAGGAAGGCTGGAAGAGG-3′

^a^ the primer sets of the TLRs seen in a previous study [42].

## Data Availability

Data are contained within the article
